# Whole-Genome Sequence of *Filimonas lacunae,* a Bacterium of the Family *Chitinophagaceae* Characterized by Marked Colony Growth under a High-CO_2_ Atmosphere

**DOI:** 10.1128/genomeA.00667-16

**Published:** 2016-07-14

**Authors:** Hatsumi Shiratori-Takano, Hideaki Takano, Kenji Ueda

**Affiliations:** Life Science Research Center, College of Bioresource Sciences, Nihon University, Fujisawa, Kanagawa, Japan

## Abstract

We report here the genome sequence of *Filimonas lacunae*, a bacterium of the family *Chitinophagaceae* characterized by high-CO_2_-dependent growth. The 7.81-Mb circular genome harbors many genes involved in carbohydrate degradation and related genetic regulation, suggesting the role of the bacterium as a carbohydrate degrader in diverse environments.

## GENOME ANNOUNCEMENT

The Gram-negative bacterial genus *Filimonas* was established based on the identification of *Filimonas lacunae* NBRC 104114, whose colony growth depends on high atmospheric CO_2_ and humidity (1–3). The genus currently consists of three species, *F. lacunae*, *Filimonas endophytica* (4), and *Filimonas zeae* (5). While *F. lacunae* was isolated from freshwater, the other two were originated from plant root. *Filimonas* constitutes a novel taxonomic group within the family *Chitinophagaceae* (6). At the time of this writing, genome sequence information from eight genera of this family is available at the NCBI database (http://www.ncbi.nlm.nih.gov/genome/). Here, we report the whole-genome sequence of *F. lacunae*, the first genome information from *Filimonas*.

Genomic DNA isolated from *F. lacunae*, as described previously (1), was checked for quality using a NanoDrop spectrophotometer (Thermo Scientific, USA) and a Qubit fluorometer (Invitrogen, USA). The genomic DNA was sheared to an average size of 10 kb using g-Tubes (Covaris, USA). A genomic library was generated using a DNA template prep kit 1.0 and DNA/polymerase binding kit P6 (Pacific Biosciences [PacBio]). Size selection and quality checking of the genome library were carried out with a BluePippin system (Nippon Genetics, Japan) and an Agilent 2100 Bioanalyzer (Agilent, USA), respectively. A PacBio RSII sequencer was used to sequence the 10-kb library of the *F. lacunae* genome using P4-C2 chemistry. Coverage of 176-fold was achieved, and the reads were assembled using the Hierarchical Genome Assembly Process version 2 (PacBio) (7). The genome sequences were successfully assembled to closure to yield a single contig. The assembled genome was then circularized prior to annotation with Rapid Annotations using Subsystems Technology (RAST) (8). The annotation was manually checked, modified, and submitted to DDBJ.

A single contig of 7,814,405 bp with 44.05% G+C content was generated from the assembly. The GC-skew profile resembled that of *Chitinophaga pinensis* (9), the close taxon of the family *Chitinophagaceae*. The genome did not contain any plasmids. RAST predicted 6,363 genes for protein-coding sequences, 15 rRNA (5 *rrn* operons), and 70 tRNA genes. The total length of these coding regions was 7,082,902 bp (90.6% of the total genome size). The majority (62.5%) of the coding regions were assigned a putative function, while those remaining were annotated as hypothetical proteins.

A whole-genome survey using the SEED viewer (10) showed the presence of a large number of genes encoding proteins involved in carbohydrate metabolism and related genetic control. Sugar-degrading enzymes affiliated with glucosidase (20 copies) and galactosidase (33 copies) and transcriptional regulators of AraC (107 copies) and cAMP receptor protein (CRP)/fumarate and nitrate reduction regulatory protein (FNR) family (45 copies) were identified. Many signal transducers, such as extracytoplasmic function (ECF)-type RNA polymerase sigma factors (86 copies), anti-sigma factors (68 copies), two-component regulatory systems (124 components), and TonB-dependent receptors (106 copies), were also identified. The genome information implies the feature of the organism as a carbohydrate degrader adapting to diverse environments.

### Nucleotide sequence accession number.

The genome sequence of *Filimonas lacunae* is available in DDBJ under the accession no. AP017422. The version described in this paper is the first version.

## References

[1] Shiratori H, Tagami Y, Morishita T, Kamihara Y, Beppu T, Ueda K 2009 *Filimonas lacunae* gen. nov., sp. nov., a member of the phylum *Bacteroidetes* isolated from fresh water. Int J Syst Evol Microbiol 59:1137–1142. doi:10.1099/ijs.0.002618-0. 19406807

[2] Ueda K, Tagami Y, Kamihara Y, Shiratori H, Takano H, Beppu T 2008 Isolation of bacteria whose growth is dependent on high levels of CO_2_ and implications of their potential diversity. Appl Environ Microbiol 74:4535–4538. doi:10.1128/AEM.00491-08. 18487395 PMC2493168

[3] Leandro T, França L, Nobre MF, Rainey FA, da Costa MS 2013 *Heliimonas saccharivorans* gen. nov., sp. nov., a member of the family *Chitinophagaceae* isolated from a mineral water aquifer, and emended description of *Filimonas lacunae*. Int J Syst Evol Microbiol 63:3793–3799. doi:10.1099/ijs.0.050021-0. 23667140

[4] Han JH, Kim TS, Joung Y, Kim SB 2015 *Filimonas endophytica* sp. nov., isolated from surface-sterilized root of *Cosmos bipinnatus*. Int J Syst Evol Microbiol 65:4863–4867. doi:10.1099/ijsem.0.000661. 26443022

[5] Gao JL, Sun P, Wang XM, Qiu TL, Lv FY, Yang MM, Lu M, Sun JG 2016 *Filimonas zeae* sp. nov., an endophytic bacterium isolated from maize root. Int J Syst Evol Microbiol, in press. doi:10.1099/ijsem.0.001116. 27118116

[6] Kämpfer P, Lodders N, Falsen E 2011 *Hydrotalea flava* gen. nov., sp. nov., a new member of the phylum *Bacteroidetes* and allocation of the genera *Chitinophaga*, *Sediminibacterium*, *Lacibacter*, *Flavihumibacter*, *Flavisolibacter*, *Niabella*, *Niastella*, *Segetibacter*, *Parasegetibacter*, *Terrimonas*, *Ferruginibacter*, *Filimonas* and *Hydrotalea* to the family *Chitinophagaceae* fam. nov. Int J Syst Evol Microbiol 61:518–523. doi:10.1099/ijs.0.023002-0. 20382796

[7] Chin C-S, Alexander DH, Marks P, Klammer AA, Drake J, Heiner C, Clum A, Copeland A, Huddleston J, Eichler EE, Turner SW, Korlach J 2013 Nonhybrid, finished microbial genome assemblies from long-read SMRT sequencing data. Nat Methods 10:563–569. doi:10.1038/nmeth.2474. 23644548

[8] Aziz RK, Bartels D, Best AA, DeJongh M, Disz T, Edwards RA, Formsma K, Gerdes S, Glass EM, Kubal M, Meyer F, Olsen GJ, Olson R, Osterman AL, Overbeek RA, McNeil LK, Paarmann D, Paczian T, Parrello B, Pusch GD 2008 The RAST server: Rapid Annotations using Subsystems Technology. BMC Genomics 9:75. doi:10.1186/1471-2164-9-75. 18261238 PMC2265698

[9] Glavina Del Rio T, Abt B, Spring S, Lapidus A, Nolan M, Tice H, Copeland A, Cheng JF, Chen F, Bruce D, Goodwin L, Pitluck S, Ivanova N, Mavromatis K, Mikhailova N, Pati A, Chen A, Palaniappan K, Land M, Hauser L 2010 Complete genome sequence of *Chitinophaga pinensis* type strain (UQM 2034). Stand Genomic Sci 2:87–95. doi:10.4056/sigs.661199. 21304681 PMC3035255

[10] Overbeek R, Olson R, Pusch GD, Olsen GJ, Davis JJ, Disz T, Edwards RA, Gerdes S, Parrello B, Shukla M, Vonstein V, Wattam AR, Xia F, Stevens R 2014 The SEED and the rapid annotation of microbial genomes using subsystems technology (RAST). Nucleic Acids Res 42:D206–D214. doi:10.1093/nar/gkt1226. 24293654 PMC3965101

